# Zinc Finger Protein 148 Is Dispensable for Primitive and Definitive Hematopoiesis in Mice

**DOI:** 10.1371/journal.pone.0070022

**Published:** 2013-07-31

**Authors:** Anna Nilton, Volkan I. Sayin, Anna Staffas, Erik Larsson, Julia Rolf, Marleen M. Petit, Lars Palmqvist, Birgitta Swolin, Susanna Cardell, Per Lindahl

**Affiliations:** 1 Wallenberg Laboratory, Institute of Medicine, Sahlgrenska Academy at the University of Gothenburg, Gothenburg, Sweden; 2 Department of Medical Biochemistry and Cell Biology, Institute of Biomedicine, Sahlgrenska Academy at the University of Gothenburg, Gothenburg, Sweden; 3 Department of Clinical Chemistry and Transfusion Medicine, Institute of Biomedicine, Sahlgrenska Academy at the University of Gothenburg, Gothenburg, Sweden; 4 Department of Microbiology and Immunology, Institute of Biomedicine, Sahlgrenska Academy at the University of Gothenburg, Gothenburg, Sweden; New York University, United States of America

## Abstract

Hematopoiesis is regulated by transcription factors that induce cell fate and differentiation in hematopoietic stem cells into fully differentiated hematopoietic cell types. The transcription factor zinc finger protein 148 (Zfp148) interacts with the hematopoietic transcription factor Gata1 and has been implicated to play an important role in primitive and definitive hematopoiesis in zebra fish and mouse chimeras. We have recently created a gene-trap knockout mouse model deficient for *Zfp148*, opening up for analyses of hematopoiesis in a conventional loss-of-function model *in vivo*. Here, we show that *Zfp148*-deficient neonatal and adult mice have normal or slightly increased levels of hemoglobin, hematocrit, platelets and white blood cells, compared to wild type controls. Hematopoietic lineages in bone marrow, thymus and spleen from *Zfp148* **^*gt/gt*^** mice were further investigated by flow cytometry. There were no differences in T-cells (CD4 and CD8 single positive cells, CD4 and CD8 double negative/positive cells) in either organ. However, the fraction of CD69- and B220-positive cells among lymphocytes in spleen was slightly lower at postnatal day 14 in *Zfp148* ^gt/gt^ mice compared to wild type mice. Our results demonstrate that *Zfp148*-deficient mice generate normal mature hematopoietic populations thus challenging earlier studies indicating that Zfp148 plays a critical role during hematopoietic development.

## Introduction

Blood formation or hematopoiesis, occurs in two distinct steps during vertebrate development, the primitive phase in the blood islands of the yolk sac and the definitive phase in the embryo proper. Hematopoiesis is regulated by general and specific transcription factors that induce cell fate and differentiation towards fully differentiated hematopoietic cell types. The discovery of new transcription factors and pathways involved in hematopoiesis may lead to the identification of novel drugable targets for the treatment of different forms of blood pathologies.

The transcription factor Zinc finger protein 148 (Zfp148; other aliases: Zbp-89, BFCOL1, BERF-1, htβ) belongs to a class of Krüppel-like transcription factors and can function both as a transcriptional activator and repressor of its target genes [1–8].

Zfp148 has been implicated to play an important role in hematopoiesis. Knockdown of the orthologue *Zbp-89* in zebrafish embryos results in a bloodless phenotype where both primitive and definitive hematopoiesis is disrupted [9]. Expression of primitive erythroid markers (gata-1 and tif1g) and primitive myeloid lineage markers (pu.1, mpo and l-plastin) were severely downregulated in *Zbp-89* morphants and it was suggested that *Zbp-89* controls hematopoiesis by acting upstream of Scl/Tal1 and Gata1. Another loss-of-function study in zebrafish suggests a role for *Zbp-89* in megakaryopoiesis and definitive but not primitive erythropoiesis [10]. Ectopic expression of *Zfp148* in zebrafish embryos conversely lead to increased expression of hematopoietic markers supporting the data from the loss-of-function experiments [9].

In mammals, Zfp148 has been identified as a novel component of the GATA1 and FOG1 multiprotein complex that regulates erythroid maturation, and Zfp148 protein was found to occupy and regulate cis-regulatory elements in globin and other erythroid-specific genes [10,11]. Experiments using mouse embryonic stem (ES) cells homozygous for a gene-trap mutation in Zfp148 further showed that Zfp148 is required for erythroid and megakaryocyte terminal maturation *in vitro*, and for contribution of ES-cells to erythroid and megakaryocyte lineages in mouse chimeras [10]. Moreover, the chimeric mice showed a reduced contribution of Zfp148-targeted stem cells to production of peripheral blood hemoglobin relative to the degree of overall hematopoietic chimerism, suggesting that Zfp148 is particularly important for erythroid differentiation. Consistent with a role in hematopoiesis, overexpression of Zfp148 in mouse ES-cells leads to increased number of hematopoietic progenitors as well as more specialized hematopoietic cell types in embryoid bodies [9].

Although there are some discrepancies between these studies, they collectively suggest that Zfp148 plays an important role in hematopoietic development. However, data on hematopoiesis in conventional knockout mice lacking Zfp148 have not been presented yet, leaving the question if Zfp148 is required for primitive or definitive hematopoiesis in mammals unanswered.

We recently generated *Zfp148*-deficient mice from a gene trap embryonic stem cell line and showed that *Zfp148* mRNA is reduced by >95% and the protein is undetectable in *Zfp148* **^*gt/gt*^** mice that are homozygous for the targeted allele [12]. *Zfp148* deficiency leads to respiratory distress in newborn mice and partial neonatal lethality (50% of the *Zfp148* **^*gt/gt*^** mice) that is caused by p53-dependent proliferation arrest of pulmonary cells and disruption of prenatal lung maturation. Homozygous *Zfp148* **^*gt/gt*^** mice that survive the neonatal crisis are growth retarded and exhibit a reduced life span.

Our results show that Zfp148 is not required for early embryonic development [12], thus raising questions regarding the importance of Zfp148 for primitive and definitive hematopoiesis in mice. Here, we specifically investigated hematopoietic lineages in peripheral blood, bone marrow, thymus and spleen from *Zfp148* **^*gt/gt*^** mice. The results demonstrate that Zfp148 is not required for primitive and definitive hematopoiesis.

## Materials and Methods

### Ethics statement

All animal experiments were approved by the animal research ethics committee in Gothenburg, Sweden (Permit Number: 225-10).

### Mice

*Zfp148* **^*+/gt*^** mice were generated from ES cell clone XB878 (BayGenomics) and maintained on a 129P2/OlaHsd and C57Bl/6 mixed genetic background, as described in [12]. PCR primers used for genotyping: GGCCCGTCATAATTTAGGTTG (forward strand) and TGCTGAGGATGAGGGAGCAG (reverse strand, *Zfp148* **^*gt*^**) and ACCGGAAGAAAAAGCAGA (reverse strand, *Zfp148* ^*wt*^). All animal experiments were approved by the animal research ethics committee in Gothenburg, Sweden (Permit Number: 225-10).

### Blood analyses and differential count

Peripheral blood drawn from the tail vein of adult mice (ages 3-16 months) and from decapitated P1 pups were analysed. Blood was transferred to EDTA-coated Microvette tubes (Sarstedt) and analysed on a Hemato analyser KX21N (Sysmex). Differential count was performed on May-Grünwald-Giemsa-stained blood smears.

### Real-time quantitative RT-PCR

Real-time quantitative RT-PCR was performed using SYBR Green PCR master mix (Applied Biosystems) and the following primers; Pu.1 forward primer: 5’-ATGCACGTCCTCGATACTCC-3’, Pu.1 reverse primer: 5’-CTCCAAGCCATCAGCTTCTC-3’ (product size: 184 bp); GATA-1 forward primer: 5’-TTCTTCCACTTCCCCAAATG-3’, GATA-1 reverse primer: 5’-GTTGAGGCAGGGTAGAGTGC-3’ (product size: 189 bp); Hbb-bh1 forward primer: 5’-TGGACAACCTCAAGGAGACC-3’, Hbb-bh1 reverse primer: 5’- CCACTCCAATCACCAGCTTC-3’ (product size: 178 bp); Gapdh exon4 forward primer: 5’-AATGTGTCCGTCGTGGATCT-3’, Gapdh exon6 reverse primer: 5’-CCCTGTTGCTGTAGCCGTAT-3’ (product size: 256 bp), Zfp148 forward primer: 5’-TGGTTTGGAAATTTAGCCAAG-’ 3, Zfp148 reverse primer: 5’-CTTCAAGGAAGGGAATGCTG 3’ (product size: 173 bp)

### Flow cytometry

Hematopoietic cell types within spleen, thymus, and bone marrow were determined using FACS. Thymus, spleen and bone marrow were dissected from 2 weeks, and 3-6 months, respectively, old mice and single-cell suspensions were prepared by passing tissue through 40µm filters and incubating in PBS with 2mM EDTA. Bone marrow and spleen samples were treated with Red Cell Lysis (Sigma) according to manufacturer’s recommendation, to reduce erythrocyte population. Isolated cells (5 × 10^6^ cells/sample) were washed once in PBS with 0,1% bovine serum albumine, blocked with Fc-block (CD16/CD32; Becton Dickinson Biosciences) at a 1:100 dilution and incubated on ice in the presence of specific monoclonal antibodies for 20 min in the dark. Labeled antibodies against the following hematopoietic cell surface markers were purchased from Becton Dickinson Biosciences; CD4 (V450), CD8a (APC-Cy7), B220 (APC), Gr1 (PE-Cy7), CD3e (V450), Ter119 (APC), CD45 (APC-Cy7), CD69 (PE-Cy7), CD62L (FITC) and CD11b/Mac1 (V450). 7AAD was used as a viability stain. Expression of cell surface markers was detected on a FACSAria flow cytometer (Becton Dickinson Biosciences) and data were analyzed using Gatelogic (eBiosciences) and BD FACS Diva (Becton Dickinson Biosciences) software. Lymphocyte gate was based on forward and side scatter intensity.

### Statistics

Values are expressed as mean ± SEM. Statistics was performed with two-tailed Student’s t-test and Kruskal-Wallis non-parametric test with Dunn’s post-hoc test. Significance levels: *P<0.05, **P<0.01.

## Results

### Primitive and embryonic hematopoiesis in *Zfp148*-deficient mice

**Figure 1:**
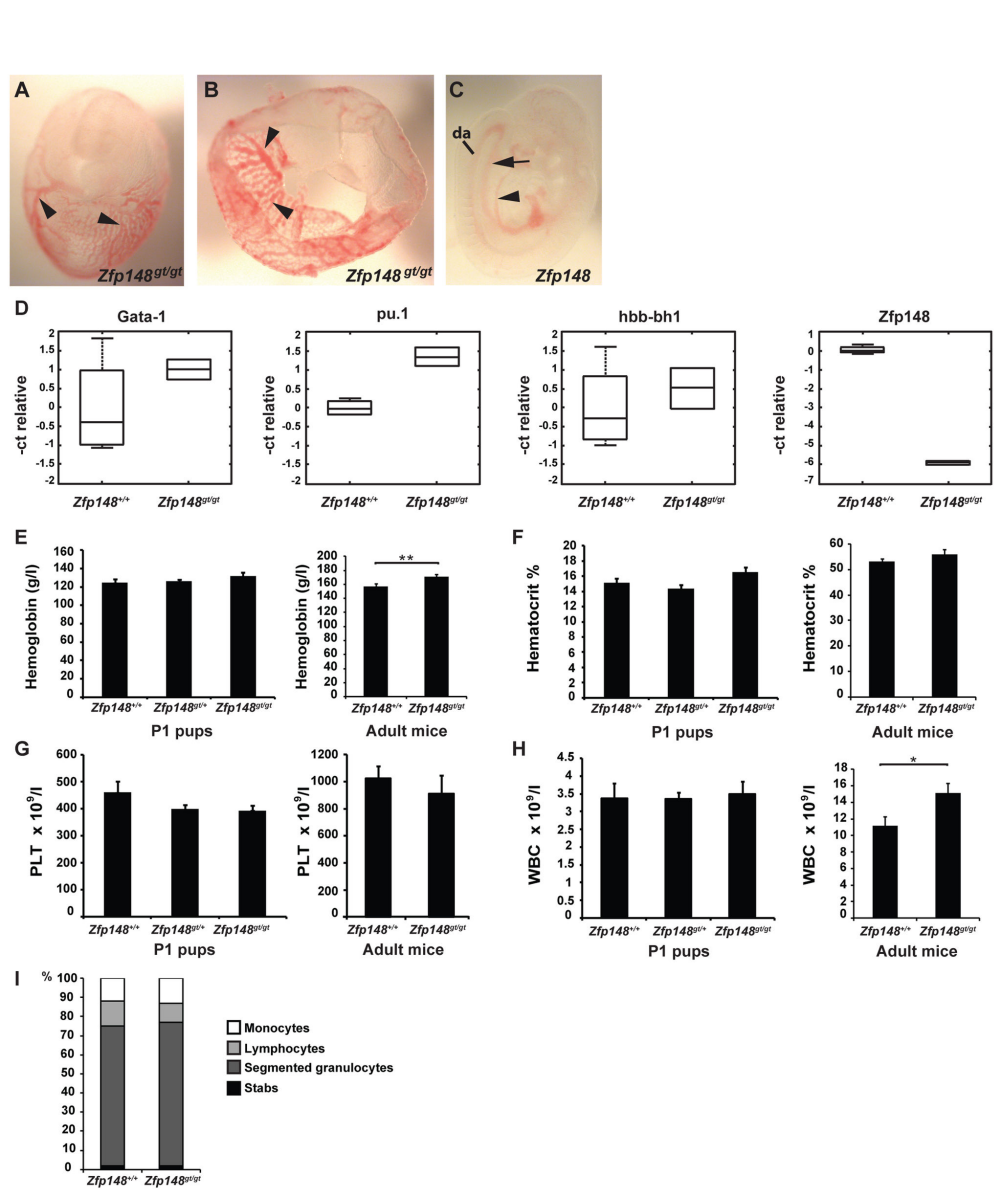
Primitive and embryonic hematopoiesis in *Zfp148*-deficient embryos and mice. (A–C) Blood filled vessels in E9.5 *Zfp148* ^***gt/gt***^ embryo and yolk sac. (A) Embryo in yolk sac. (B) Primitive vascular plexa in *Zfp148* ^***gt/gt***^ yolk sac (arrowheads). (C) Apparent presence of major blood vessels in *Zfp148* ^***gt/gt***^ embryo proper (da: dorsal aorta; arrow indicating posterior cardinal vein; arrowhead indicating anterior cardinal vein). (D) Real-time RT-PCR showing relative levels of biomarkers of hematopoietic differentiation. The edges of the boxes show the 25th and 75th percentiles, the central mark is the median, and the whiskers extend to the most extreme data points. (E–H) Blood parameters in peripheral blood from postnatal day 1 pups (P1, left graph) and adult mice (right graph; ages 2-12 months). (E) Hemoglobin, (F) hematocrit and (G) number of platelets in P1 pups; wild type (*n* = 21), *Zfp148* ^***gt/+***^ (*n* = 36) and *Zfp148* ^***gt/gt***^ (*n* = 6-7) and adult mice; wild type (*n* = 19) and *Zfp148* ^***gt/gt***^ (*n* = 15). (H) White blood cells in P1 pups; wild type (*n* = 7), *Zfp148* ^***gt/+***^ (*n* = 32) and *Zfp148* ^***gt/gt***^ (*n* = 6). Adult mice; wild type (*n* = 19) and *Zfp148* ^***gt/gt***^ (*n* = 13). (I) Differential count of blood smears from P1 mice. Percentage of leukocyte populations; monocytes, lymphocytes, segmented granulocytes and stabs in wild type (*n* = 18), *Zfp148* ^***gt/+***^ (*n* = 15) and *Zfp148* ^***gt/gt***^ (*n* = 11). Values are expressed as mean ± SEM. Significance levels: *P<0.05, **P<0.01.

To investigate the role of Zfp148 in primitive and definitive hematopoiesis, Zfp148^+/+^ and *Zfp148* ^gt/gt^ embryos at embryonic day 9.5 (E9.5) were dissected. Blood vessels and erythrocytes were present in yolk sac as well as in embryo proper (Figure 1A–C). Quantitative real-time PCR analysis showed normal or elevated expression of the hematopoietic transcription factor GATA-1, β-hemoglobin and pu.1 in yolk sacs from *Zfp148* **^gt/gt^** embryos compared to wild type (Figure 1D). Those data together with the lack of embryonic lethality of *Zfp148* **^gt/gt^** embryos [12] strongly suggest that Zfp148 is not required for primitive and definitive hematopoiesis.

### Postnatal and adult hematopoiesis in Zfp148-deficient mice

Half of the homozygous *Zfp148* **^*gt/gt*^** mice die shortly after birth due to a lung maturation defect [12]. *Zfp148* **^*gt/gt*^** mice that survive the neonatal crisis are growth retarded and exhibit a reduced life span, but otherwise appear grossly normal.

To assess hematopoiesis in postnatal *Zfp148* ^*gt/gt*^ mice, peripheral blood was collected from decapitated pups. There were no alterations in hemoglobin, hematocrit, platelets or white blood cell (WBC) count compared to wild type mice at P1 (Figure 1E–H). In peripheral blood drawn from the tail vein of adult (2-12 months old) *Zfp148* **^gt/gt^** mice there was a slight elevation of hemoglobin compared to wild type (*P*=0.003, Figure 1E) but no significant differences in hematocrit values or platelet counts (Figure 1F, G). Adult *Zfp148* **^gt/gt^** mice also displayed a higher WBC count (*P*=0.03, Figure 1H) in peripheral blood.

To assess the distribution of leukocyte subsets, we performed a differential count on blood smears from *Zfp148* ^*+/+*^ and *Zfp148* **^gt/gt^** mice at P1. There were no differences in percentage of monocytes, lymphocytes, segmental granulocytes or stabs among leukocytes (Figure 1I). In all, these data show that *Zfp148* is not required for erythropoiesis, myelopoiesis and megakaryopoiesis in mice. Instead, we observe an increase in both hemoglobin and WBC in peripheral blood of adult *Zfp148*-deficient mice.

### Differentiation of hematopoietic lineages

Primary lymphoid organs, such as bone marrow and thymus, are the sites of lymphocyte generation and maturation. In secondary lymphoid organs, such as the spleen, lymphocytes undergo maturation, selection and expansion. In order to investigate the role of *Zfp148* in hematopoietic differentiation as well as maturation and activation of lymphocytes, we isolated bone marrow from adult mice and thymocytes and splenocytes from postnatal day 14 (P14) and adult mice (ages 3-6 months) for flow cytometric analyses.

**Figure 2:**
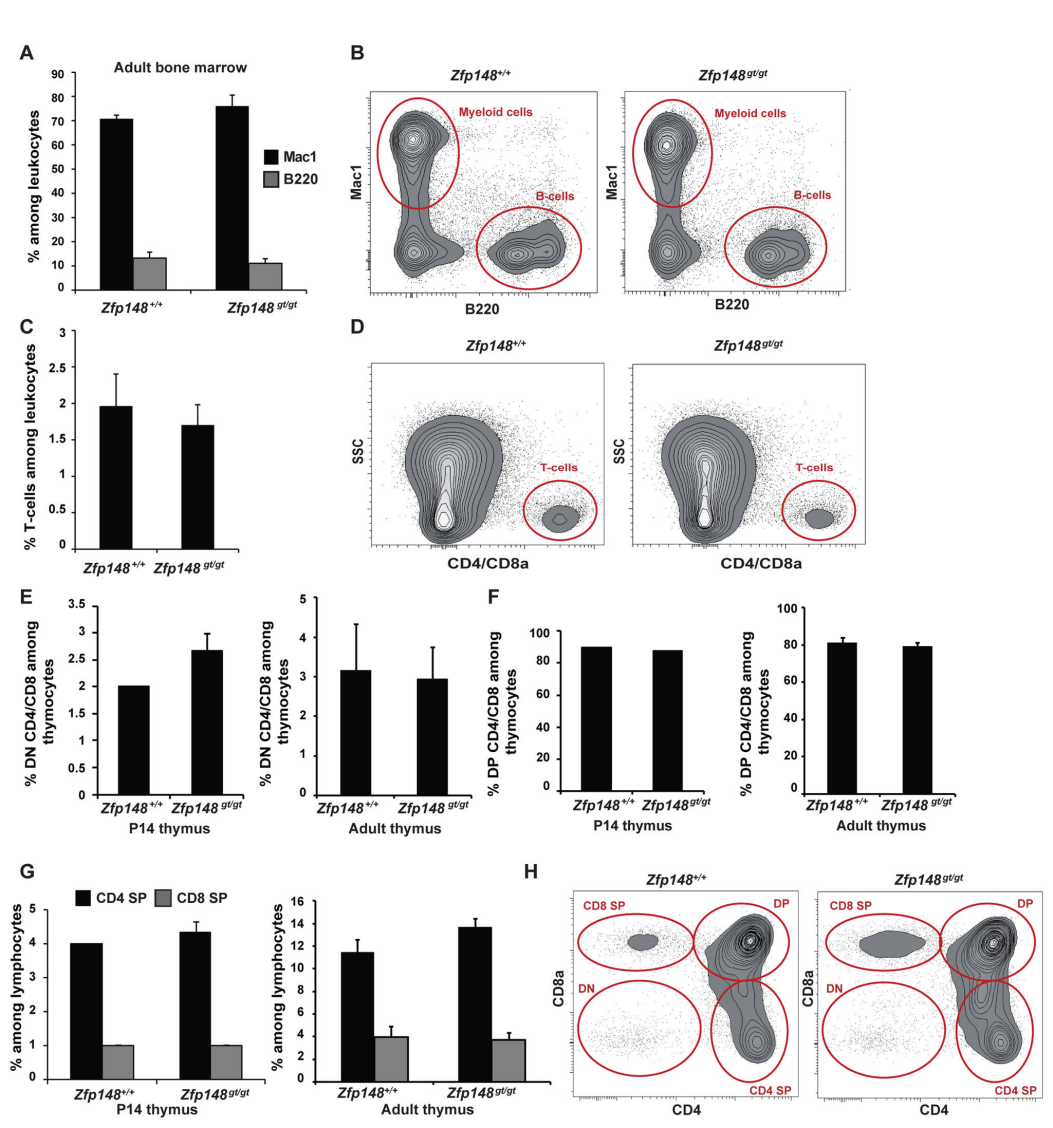
Differentiation of hematopoietic lineages in thymus and bone marrow of *Zfp148*-deficient mice. Flow cytometric analysis of bone marrow (A–D) and thymocytes (E–H) from wild type and *Zfp148* ^*gt/gt*^ mice. Graphs and representative cytograms show percentage of (A, B) Mac1-, B220- and (C, D) CD4 and/or CD8-positive cells among leukocytes in bone marrow from adult mice, ages 3-6 months; wild type (*n* = 6) and *Zfp148* **^*gt/gt*^** (*n* = 5). (E–H) Percentage of (E) double negative (DN) CD4/CD8, (F) double positive (DP) CD4/CD8, (G) CD4 single positive (SP) and CD8 SP cells among thymocytes from P14 pups (left, *n* = 3) and adult mice (right, *n* = 5). (H) Representative cytograms from adult thymus. Values are expressed as mean ± SEM. Significance levels: *P<0.05, **P<0.01.

Cells isolated from bone marrow of *Zfp148* **^gt/gt^** mice did not differ regarding expression of the myeloid marker Mac-1 (CD11b/CD18), the B-cell marker B220 nor the T-cell markers CD4/CD8, compared to wild type mice (Figure 2A–D). In thymus, immature T-cell populations are CD4 and CD8 double negative, these will later on differentiate to CD4 and CD8 double positive cells and mature into either T-helper cells (CD4 single positive) or cytotoxic T-cells (CD8 single positive). There were no differences between *Zfp148* **^gt/gt^** and wild type mice in the proportions of double negative (DN; Figure 2E, H), double positive CD4/CD8 (DP; Figure 2F, H) or single positive cells (SP, Figure 2G, H) among thymocytes at either age. Furthermore, the more rare populations of invariant natural killer T-cells (positive for α-galactosylceramide loaded DimerX) or TCRγδ T-cells did not differ between the genotypes in P14 thymus (data not shown). Taken together, these results suggest that Zfp148 is not essential for hematopoiesis in primary lymphoid organs.

**Figure 3:**
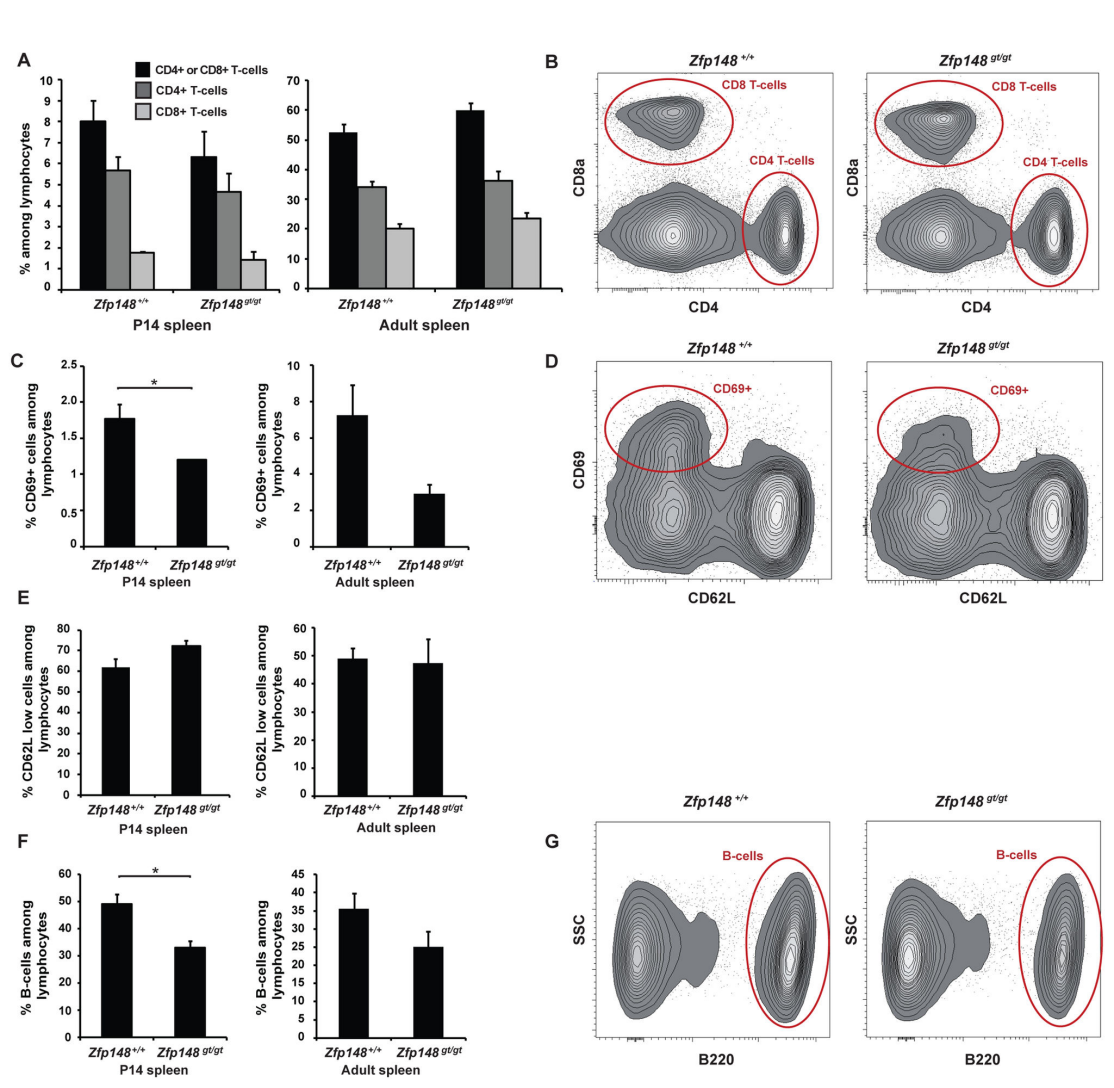
Differentiation of hematopoietic lineages in spleen of *Zfp148*-deficient mice. Flow cytometric analysis of spleen from P14 pups (*n* = 3) and adult mice, ages 3-6 months; wild type (*n* = 6) and *Zfp148* **^*gt/gt*^** (*n* = 5). (A) Percentage of CD4- or CD8-positive T-cells, CD4-positive T-cells and CD8-positive T-cells among lymphocytes. (B) Representative cytograms from adult spleens. (C) Percentage of CD69-positive cells among lymphocytes. (D) Representative cytograms from adult spleens. (E) CD62L low expressing cells among lymphocytes. (F) Percentage of B220-positive cells among lymphocytes in P14 and adult spleen. (G) Representative cytograms from adult spleens. Lymphocytes were gated based on side scatter (SSC). Values are expressed as mean ± SEM. Significance levels: *P<0.05, **P<0.01.

Next, we assessed the differentiation of lymphocyte lineages in a peripheral lymphoid organ, i.e. the spleen of *Zfp148* **^gt/gt^** and wild type mice. There was no difference between the genotypes in the percentage of CD4 positive and/or CD8 positive T-cells (Figure 3A, B) among lymphocytes in spleens at either age. An immune response is initiated upon T-cell recognition of its corresponding antigen. Subsequently, this leads to activation of T-cells with up-regulation of surface markers such as the C-type lectin CD69 and down-regulation of others, such as the L-selectin (CD62L). Spleens from *Zfp148* ^*gt/gt*^ mice exhibited lower percentage of CD69-positive cells among lymphocytes compared to wild type at age P14 (Figure 3C, D) and a tendency towards lower percentage in adult mice (*P*=0.07, Figure 3C, D). However, there were no differences in the percentage of CD62L low expressing cells among lymphocytes from *Zfp148* **^gt/gt^** and wild type spleens at either age (Figure 3D, E).

Immature, short-lived B-cell precursors are formed in the bone marrow and migrate to secondary lymphoid organs such as the spleen where some of these so called transitional B-cells mature into long-lived B-lymphocytes [13]. From early pro-B stages and onwards, B-cells express an isoform of CD45R called B220 which is down-regulated upon terminal differentiation to plasma cells. The fraction of B-cells among lymphocytes in spleen (as assessed with antibodies directed against B220/CD45R) was decreased by 33% in P14 *Zfp148* **^*gt/gt*^** mice compared to wild type mice (*P*=0.02, Figure 3F, G). There was no significant difference in adult mice (*P*=0.15, Figure 3F, G).

## Discussion

In summary, we show that *Zfp148* is not required for primitive and definitive hematopoiesis in mice. All investigated hematopoietic cell lineages were present in equal or elevated levels in blood, bone marrow or thymus from *Zfp148* **^*gt/gt*^** mice, compared to wild type mice. There were, however, lower percentages of CD69-positive T-cells and B220-positive B-cells among lymphocytes in spleens of *Zfp148* ^gt/gt^ mice, suggesting a possible role for Zfp148 in lymphocyte activation or maturation in peripheral lymphoid organs.

Half of the *Zfp148* **^*gt/gt*^** mice die shortly after birth due to a lung maturation defect. It is well known that lung disorders in humans may lead to hypoxemia and secondary polycythemia, opening up for the possibility that hypoxemia could mask deficient erythropoiesis in *Zfp148* ^gt/gt^ mice. However, the erythrocyte counts in peripheral blood on postnatal-day 1 were performed less than 12 hours after birth of the *Zfp148*-deficient mice and controls. We find it unlikely that compensatory polycythemia would appear in such a short timeframe. Moreover, the lung phenotype of newborn mice is not present in adult mice showing that the lung tissue heals in *Zfp148*-deficient pups that survive the neonatal crisis (Supporting Information Figure S1). Importantly, adult *Zfp148* ^*gt/gt*^ mice did not display lower than normal erythrocyte counts.

Clearly, our results differ from the previously published study on the role of *Zfp148* in definitive hematopoiesis in mouse chimeras [10]. *Zfp148* mRNA levels are reduced >95% in the *Zfp148* **^gt/gt^** mouse model compared to wild-type, as judged by real-time RT-PCR analyses of mRNA from yolk sac (Figure 1D), liver, fat, muscle and hypothalamus (see Figure 1D in [12]), and Zfp148 protein is undetectable (See Figure 1E in [12]). Persistent hematopoiesis in *Zfp148* **^*gt/gt*^** mice is therefore not explained by inefficient gene targeting. Moreover, the mouse chimeras studied by Woo et al. [10], and the *Zfp148* **^*gt/gt*^** mice used in our study, were generated from the same gene-trap embryonic stem cell line.

In mouse chimeras, progeny of the injected stem cells compete with blastocyst-derived cells for representation in the various cell lineages of the mouse [14]. The method is highly sensitive and may reveal gene functions that are not detected by conventional targeting strategies. For example, *Pdgfrb*-deficient embryonic stem cells do not contribute to skeletal muscle lineages when competing with wild type cells in mouse chimeras indicating that *Pdgfrb* plays an important role in skeletal muscle differentiation. However, no skeletal muscle defects are reported in *Pdgfrb* ^*-/-*^ embryos [15]. Hematopoietic defects in *Zfp148* ^*gt/gt*^ mice could therefore be masked by homeostatic effects.

Another possible explanation for the divergent results is that the embryonic stem cell line used to generate the mouse chimeras harbors gene-trap insertions in other genes than *Zfp148*. During our generation of the *Zfp148* ^*gt/gt*^ mouse model, we discovered that offspring from the first generation (F1) intercrosses of those mice displayed a lethal embryonic phenotype that segregated with the gene trap vector, but not with the recombined *Zfp148* allele [12]. Thus, the hematopoietic defects in the mouse chimeras, generated from the same ES cell clone, could result from the insertion of a second gene-trap vector and disruption of genes other than *Zfp148*.

A critical role for Zfp148 in hematopoietic development is supported by two independent studies showing hematopoietic phenotypes in zebrafish *Zbp-89* morphants [9,10]. However, overt phenotypic discrepancies raise questions regarding the interpretation of those results. In one study, knockdown of *Zbp-89* blocks primitive and definitive hematopoiesis causing a blood less phenotype [9]. In contrast, the other study suggests that *Zbp-89* is dispensable for primitive hematopoiesis but required for megakaryocyte differentiation [10]. Until these inconsistencies have been sorted out, the role of *Zbp-89* in zebrafish development remains unclear.

Although our study shows that Zfp148 is not required for hematopoietic development in mice, the results do not exclude the possibility that Zfp148 plays an important role in hematopoiesis. Genetic targeting of *Flt3*, which is an established regulator of hematopoietic cells, similarly generated mice with normal mature hematopoietic populations [16]. The fraction of B220 positive B-cells among lymphocytes was reduced in spleens, but not in bone marrow, of P14 *Zfp148* **^gt/gt^** mice compared to wild type. Similarly, the fraction of CD69-positive cells among lymphocytes was reduced in *Zfp148* ^*gt/gt*^ spleens. This might suggest that Zfp148 plays a role during activation or expansion of mature T- and B-cell populations that occurs in secondary lymphoid organs such as the spleen. Moreover, the increase in hemoglobin and WBC count in peripheral blood from adult *Zfp148* **^gt/gt^** mice indicate that Zfp148 may be involved in the fine-tuning of erythroid or myeloid lineages. Studies of the exact role of Zfp148 in those processes will however require conditional targeting of *Zfp148* in specific hematopoietic lineages to exclude the possibility of compensatory changes in response to global *Zfp148*-deficiency.

In conclusion, we show that *Zfp148* is dispensable for primitive and definitive hematopoiesis in mice challenging earlier reports suggesting that Zfp148 plays a critical role in those processes.

## Supporting Information

Figure S1 Lung morphology in adult *Zfp148* ^*gt/gt*^ mice and controls. No indication of lung maturation defects in adult *Zfp148* ^*gt/gt*^ mice. Hematoxylin and eosin staining of 5µm paraffin section of inflation fixed lung tissue from two *Zfp148* ^*gt/gt*^ mice and wild type (wt) controls, respectively, at age 4 months. Scale bars, 200µm.
(TIF)
