# Supplementary figures and images for: Zinc Finger Protein 148 Is Dispensable for Primitive and Definitive Hematopoiesis in Mice
Source: PLoS One. 2013 Jul 31;8(7):e70022. doi: 10.1371/journal.pone.0070022 (PMC3729454; doi:10.1371/journal.pone.0070022)

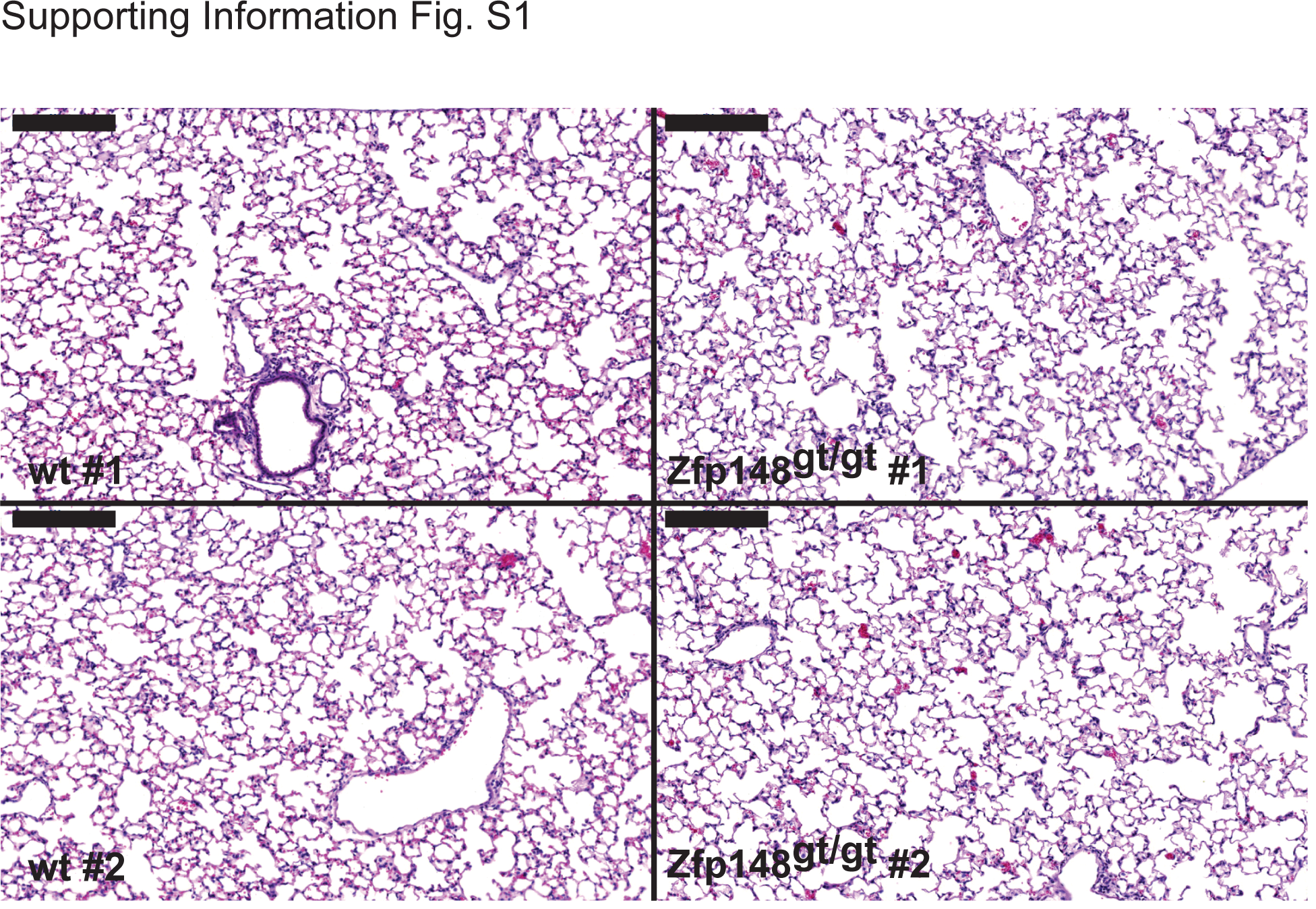

Supplement: Figure S1 — No indication of lung maturation defects in adult Zfp148 gt/gt mice. Hematoxylin and eosin staining of 5µm paraffin section of inflation fixed lung tissue from two Zfp148 gt/gt mice and wild type (wt) controls, respectively, at age 4 months. Scale bars, 200µm. (TIF) [file pone.0070022.s001.tif]
